# Diagnostic performance of CT with Valsalva maneuver for the diagnosis and characterization of inguinal hernias

**DOI:** 10.1007/s10029-023-02830-y

**Published:** 2023-07-06

**Authors:** S. Ghafoor, A. Tognella, D. Stocker, A. M. Hötker, M. Kaniewska, T. Sartoretti, A. Euler, R. Vonlanthen, M. Bueter, H. Alkadhi

**Affiliations:** 1 Diagnostic and Interventional Radiology, University Hospital Zurich, University of Zurich, Zurich, Switzerland; 2 Department of Visceral and Transplantation Surgery, University Hospital of Zurich, Zurich, Switzerland

**Keywords:** Hernia, Inguinal, Multidetector computed tomography, Valsalva Maneuver, Diagnostic imaging

## Abstract

**Purpose:**

Inguinal hernias are mainly diagnosed clinically, but imaging can aid in equivocal cases or for treatment planning. The purpose of this study was to evaluate the diagnostic performance of CT with Valsalva maneuver for the diagnosis and characterization of inguinal hernias.

**Methods:**

This single-center retrospective study reviewed all consecutive Valsalva-CT studies between 2018 and 2019. A composite clinical reference standard including surgery was used. Three blinded, independent readers (readers 1–3) reviewed the CT images and scored the presence and type of inguinal hernia. A fourth reader measured hernia size. Interreader agreement was quantified with Krippendorff’s α coefficients. Sensitivity, specificity, and accuracy of Valsalva-CT for the detection of inguinal hernias was computed for each reader.

**Results:**

The final study population included 351 patients (99 women) with median age 52.2 years (interquartile range (IQR), 47.2, 68.9). A total of 381 inguinal hernias were present in 221 patients. Sensitivity, specificity, and accuracy were 85.8%, 98.1%, and 91.5% for reader 1, 72.7%, 92.5%, and 81.8% for reader 2, and 68.2%, 96.3%, and 81.1% for reader 3. Hernia neck size was significantly larger in cases correctly detected by all three readers (19.0 mm, IQR 13, 25), compared to those missed by all readers (7.0 mm, IQR, 5, 9; p < 0.001). Interreader agreement was substantial (α = 0.723) for the diagnosis of hernia and moderate (α = 0.522) for the type of hernia.

**Conclusion:**

Valsalva-CT shows very high specificity and high accuracy for the diagnosis of inguinal hernia. Sensitivity is only moderate which is associated with missed smaller hernias.

**Supplementary Information:**

The online version contains supplementary material available at 10.1007/s10029-023-02830-y.

## Introduction

Inguinal hernias are the most common form of hernias where a defect of the abdominal wall leads to protrusion of the parietal peritoneum with or without abdominal contents at the level of the groin [1]. The lifetime risk of developing inguinal hernia is estimated to be 27–43% in men and 3–6% in women with increasing incidence at advanced age and with higher body mass index [1–3]. Inguinal hernias can be complicated by incarceration or strangulation [4]. Surgical repair is the treatment of choice for symptomatic disease.

According to current guidelines, clinical assessment remains the mainstay for diagnosing inguinal hernia, and imaging is seldom warranted [5, 6]. Nonetheless, imaging can assist in diagnosing clinically occult cases and for surgical planning [7, 8]. Furthermore, differentiating hernia types and confirming bilateral hernias through clinical examination is challenging, but important for planning treatment [9, 10].

Ultrasound (US) with dynamic maneuvers (e.g., Valsalva maneuver) is most often used for the assessment of inguinal hernia [11–13]. Valsalva maneuver increases hernia conspicuity as the hernia sac protrudes more under increased abdominal pressure. Drawbacks of US are its operator-dependence and limitations related to patient size. Moreover, interpretation of US images for treatment planning purposes can be challenging for the surgeon [14, 15].

Magnetic resonance imaging (MRI) is less examiner-dependent and images are easier to interpret for non-radiologists given the possibility of multiplanar views [16]. However, MRI has some contraindications (claustrophobia, non-compatible devices) and may not be readily available at every institution.

The literature about the accuracy of computed tomography (CT) imaging for the diagnosis of inguinal hernia is scarce. One study evaluated CT under Valsalva maneuver (Valsalva-CT) for the diagnosis of abdominal wall hernias [17]. However, only five inguinal hernias were included and there was no reference standard to verify findings. Others investigated the diagnostic performance of CT in prone positioning for the diagnosis of inguinal hernias and found higher accuracy of prone compared to supine CT (98.1 vs. 72.8%) [18, 19]. However, prone positioning may not be feasible for all patients and may mask the presence of other concurrent abdominal wall hernias.

We are routinely using a dedicated protocol consisting of a non-contrast CT of the abdomen/pelvis in supine position acquired during a Valsalva maneuver in patients with suspected inguinal hernia to screen for additional unsuspected occult hernias and other abdominal wall hernias as these could influence treatment planning. The primary objective of this study was to evaluate the diagnostic performance of Valsalva-CT for the diagnosis and characterization of inguinal hernias. Secondary objectives were to assess interreader agreement and to investigate influencing factors on the diagnostic performance of the modality.

## Materials and methods

The institutional review board and local ethics committee approved this retrospective study (BASEC-Nr: 2021-02464). Patients were included if they had signed a written general informed consent form for the anonymized use of their data for research.

### Patient population

**Fig. 1:**
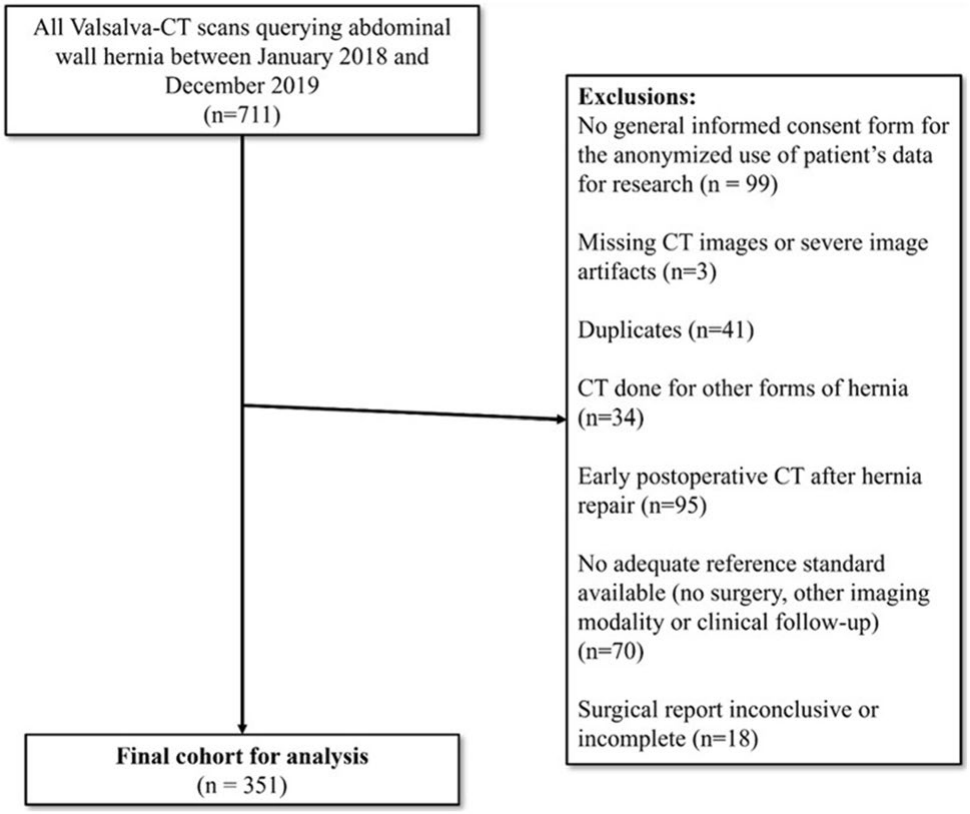
Flowchart of patient inclusion

The departmental radiology information system (RIS) was searched for all consecutive patients who had undergone a Valsalva-CT for the evaluation of abdominal wall hernias between January 2018 and December 2019. Exclusion criteria were: no signed general informed consent for research-related use of anonymized patient data, duplicates (only the initial CT was included if the same patient had more than one CT within the study period), CT images missing or degraded by image artifacts, CT performed for the evaluation of other hernia types (i.e., Bochdalek hernia), early postoperative CT, no adequate reference standard, and inconclusive surgical report (e.g., if there was no clear description of the hernia type) (Fig. 1). Demographic and clinical data were retrieved from the electronic medical records.

### Reference standard

All patients were assessed clinically by abdominal surgeons from our institution. Inguinal hernias were either diagnosed clinically by physical exam or a high suspicion was raised based on clinical findings. The indication for Valsalva-CT was to screen for the presence of abdominal wall hernias. A composite clinical reference standard was used. In those patients who had undergone surgery, the intraoperative findings were used as a reference standard. In the others, a combination of clinical examination findings according to current guidelines [5, 20], another imaging modality (either US or MRI), and clinical follow-up of at least 2 years was used as a reference standard. Abdominal surgeons from our hospital performed all surgical hernia repairs. Procedures included laparoscopic or open techniques. Details about intraoperative findings including the type of hernia (direct, indirect, combined) were retrieved from the surgical reports.

### CT imaging

All patients were scanned on a dual-source or single-source CT scanner (SOMATOM Force, SOMATOM Definition Edge, Siemens Healthineers, Forchheim, Germany). Protocol details are outlined in Supplemental Table 1. All patients received one non-contrast CT of the abdomen and pelvis in supine position and images were acquired during a maximum Valsalva maneuver. Additional images without Valsalva maneuver are not obtained in our clinical practice due to lack of added benefit [17] and considerations related to radiation exposure. Prior to obtaining the CT scan, all patients were instructed thoroughly by the CT technologists on how to perform the Valsalva maneuver correctly and were allowed to practice under guided supervision. For the time of the image acquisition, the patients were asked to bear down as much as they could, “push out their belly”, and hold their breath for as long as they could (Supplemental Figure 1). No oral or rectal contrast was given.

### Image analysis

Three board-certified radiologists with 5 (reader 1, D.S.), 8 (reader 2, A.H.), and 4 (reader 3, M.K.) years of experience in abdominal radiology reviewed the images. Reader 1 and reader 2 were radiologists with subspecialization in abdominal imaging. The readers, who were blinded to the reference standard, independently reviewed the CT images and scored for each patient and each side of the groin whether there was an inguinal hernia or not. Any fascial defect or interruption of the abdominal wall in the groin region with or without bulging fat or protrusion of intraabdominal structures was defined as a hernia. If a hernia was scored as present, readers were asked to define the type (indirect, direct, or combined). Indirect hernias originate posterolateral and superior to the course of the inferior epigastric vessels while direct hernias originate anteromedial and inferior to the course of these vessels [5, 21]. Combined hernias were defined as the occurrence of a direct and indirect hernia on the same side of the groin.

**Fig. 2:**
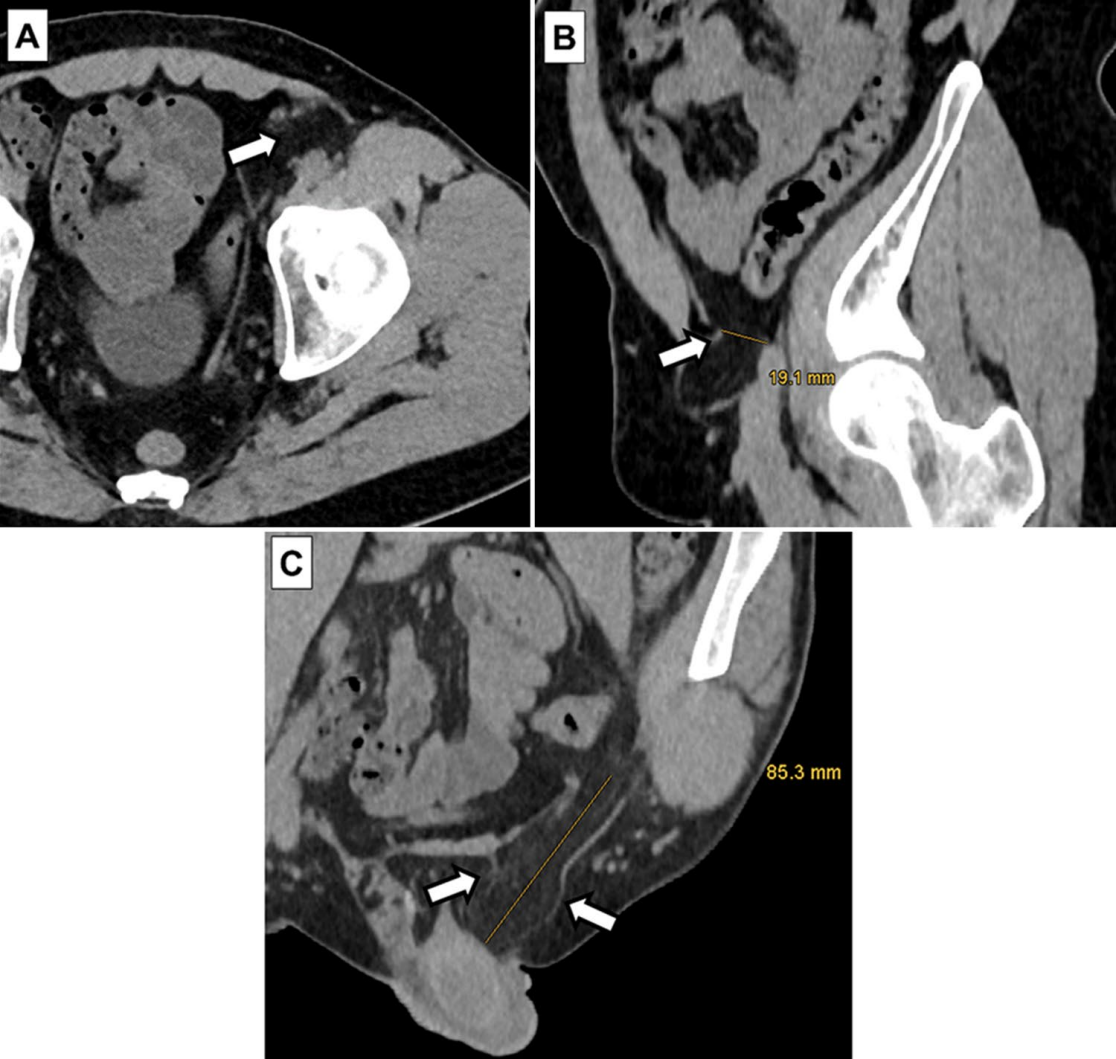
Axial (**A**), sagittal oblique (**B**) and coronal oblique (**C**) CT images of a 49-year-old male patient with a fat-containing indirect inguinal hernia on the left side. Hernia neck size (arrows in **A** and **B**) measured 19.1 mm and the maximum diameter of the hernia sac (arrows in **C**) was 85.3 mm

A fourth, unblinded reader (reader 4, S.G.) with 5 years of experience in abdominal imaging measured the hernia size using the electronic caliper tool in the PACS and recorded the hernia contents. Measurements included the size of the hernia neck and the maximum diameter of the hernia sac (Fig. 2).

### Statistical analysis

The statistical analysis was performed using R statistical software (version 4.0.2.; R Core Team, R Foundation for Statistical Computing, Vienna, Austria) and SPSS (version 26.0, IBM Corp, Armonk, NY). Clinical data and readout results were evaluated using descriptive statistics. Continuous variables are presented as either mean ± standard deviation or median (interquartile range) and categorical variables as numbers with percentages.

Diagnostic performance (sensitivity, specificity, accuracy) of Valsalva-CT for the detection of inguinal hernias was computed for each reader and for each side of the groin separately as well as for both sides together. For each test characteristic, the 95% confidence interval (CI) was determined by using the standard normal approximation of the binomial distribution table. Performance in classification of hernia type (direct, indirect, or combined) was assessed through calculation of accuracy and balanced accuracy for each reader. Interreader agreement was quantified with Krippendorff’s α coefficients (0.0–0.20 = poor agreement, 0.21–0.40 = fair agreement, 0.41–0.60 = moderate agreement, 0.61–0.80 = substantial agreement, and 0.81–1.00 = almost perfect agreement) [22] and by computing the percentage agreement. Normal distribution of data was tested with the Kolmogorov–Smirnov test. Comparisons between groups were analyzed with the Mann Whitney U test. Two-tailed p-values below 0.05 were considered to infer statistical significance.

## Results

### Patient population and reference standard

The initial search in the RIS yielded 711 eligible CT scans. Following exclusions were made: no signed general informed consent for research-related use of anonymized patient data (n = 99), duplicates (n = 41), CT images missing or degraded by image artifacts (n = 3), CT performed for the evaluation of other hernia types (n = 34), early postoperative CT (n = 95), no adequate reference standard (n = 70), and inconclusive surgical report (n = 18). After applying exclusion criteria, the final study population included 351 patients (Fig. 1).

A total of 381 inguinal hernias (right side n = 185, left side n = 196) were present in 221 patients (221/351, 63.0%). One hundred thirty patients (130/351, 37%) had no inguinal hernia. Seventy-nine patients (79/351, 22.5%) had a history of prior inguinal hernia repair. Median time in months between CT and surgery (in those patients were operated) was 2 months (1, 4). Main patient and hernia characteristics are outlined in Table 1.

**Table 1:** Patient demographics

	Total population (n = 351)	Female patients (n = 99)	Male patients (n = 252)
Age (years)	56.8 (47.2, 68.9)	52.2 (44.9, 67.0)	58.2 (48.4, 69.6)
Sex			
Male	252 (71.8%)		
Female	99 (28.2%)		
Body mass index (kg/m^2^)	25.7 (23.1, 29.0)	26.7 (22.3, 31.2)	25.7 (23.2, 28.3)
No. of patients with inguinal hernia	221/351 (63.0%)	31/99 (31.3%)	190/252 (75.3%)
No. of inguinal hernias	381	53	328
No. of patients with bilateral inguinal hernias			
Of all patients	160/351 (45.6%)	22/99 (22.2%)	138/252 (54.8%)
Of patients with inguinal hernias	160/221 (72.4%)	22/31 (71.0%)	138/190 (72.6%)
Type of inguinal hernias			
Indirect	215/381 (56.4%)	44/53 (83.0%)	171/328 (52.1%)
Direct	116/381 (30.5%)	8/53 (15.1%)	108/328 (32.9%)
Combined	50/381 (13.1%)	1/53 (1.9%)	49/328 (15.0%)
Hernia size			
All hernias			
Hernia neck (mm)	13.0 (8.3, 20.8)		
Hernia sac (mm)	37.0 (25.0, 55.0)		
Combined hernias			
Hernia neck smaller component (mm)	8.0 (6.3, 11.0)		
Hernia neck larger component (mm)	18.0 (12.0, 24.0)		
Hernia sac smaller component (mm)	17.0 (11.5, 25.5)		
Hernia sac larger component (mm)	40.0 (31.5, 63.0)		
Hernia contents			
Fat	276/381 (72.4%)	48/53 (90.6%)	228/328 (69.5%)
Bowel	93/381 (24.4%)	5/53 (9.4%)	88/328 (26.8%)
Bladder	11/381 (2.9%)	–	11/328 (3.4%)
Fluid	1/381 (0.3%)	–	1/328 (0.3%)

Data are either numbers with percentages in parentheses or medians with interquartile range in parentheses

### Diagnostic performance of Valsalva-CT for diagnosis of inguinal hernia

Sensitivity of Valsalva-CT for the diagnosis of inguinal hernia was 85.5% for reader 1, 72.7% for reader 2, and 68.2% for reader 3. Specificity was 98.1% for reader 1, 92.5% for reader 2, and 96.3% for reader 3. Accuracy was 91.5% for reader 1, and 81.8% for reader 2, and 81.1% for reader 3. The detailed overall and side-specific results are outlined in Table 2.

**Table 2:** Side-specific diagnostic performance of Valsalva-CT for diagnosis of inguinal hernia

	Right side	Left side	Both sides
Reader 1			
Sensitivity	86.4% (81.3%, 91.3%) [160/185]	85.2% (79.1%, 89.3%) [167/196]	85.8% (82.3%, 89.3%) [327/381]
Specificity	97.6% (95.3%, 99.9%) [162/166]	98.7% (94.9%, 99.9%) [153/155]	98.1% (96.6%, 99.6%) [315/321]
Accuracy	91.7% (88.3%, 94.4%) [322/351]	91.2% (86.4%, 93.0%) [320/351]	91.5% (89.1%, 93.4%) [642/702]
Reader 2			
Sensitivity	71.4% (65.1%, 78.1%) [132/185]	74.0% (67.8%, 80.1%) [145/196]	72.7% (68.2%, 77.2%) [277/381]
Specificity	95.8% (92.0%, 98.4%) [159/166]	89.0% (84.1%, 94.0%) [138/155]	92.5% (89.6%, 95.4%) [297/321]
Accuracy	82.9% (78.6%, 86.7%) [291/351]	80.6% (76.1%, 84.6%) [283/351]	81.8% (78.7%, 84.6%) [574/702]
Reader 3			
Sensitivity	68.1% (61.0%, 74.5%) [126/185]	68.4% (61.9%, 74.9%) [134/196]	68.2% (63.6%, 72.9%) [260/381]
Specificity	97.6% (94.4%, 99.6%) [162/166]	94.8% (91.4%, 98.3%) [147/155]	96.3% (94.2%, 98.3%) [309/321]
Accuracy	82.1% (77.3%, 85.7%) [351]	80.1% (75.5%, 84.1%) [281/351]	81.1% (78.0%, 83.9%) [569/702]

Numbers in parentheses represent 95% confidence intervals

Numbers in square brackets represent total numbers

Results for the diagnostic performance of Valsalva-CT for detection of bowel- or bladder-containing hernias and for cases with prior inguinal hernia repair are outlined in Supplemental Text 1.

### Diagnostic performance of Valsalva-CT for the type of inguinal hernia

**Fig. 3:**
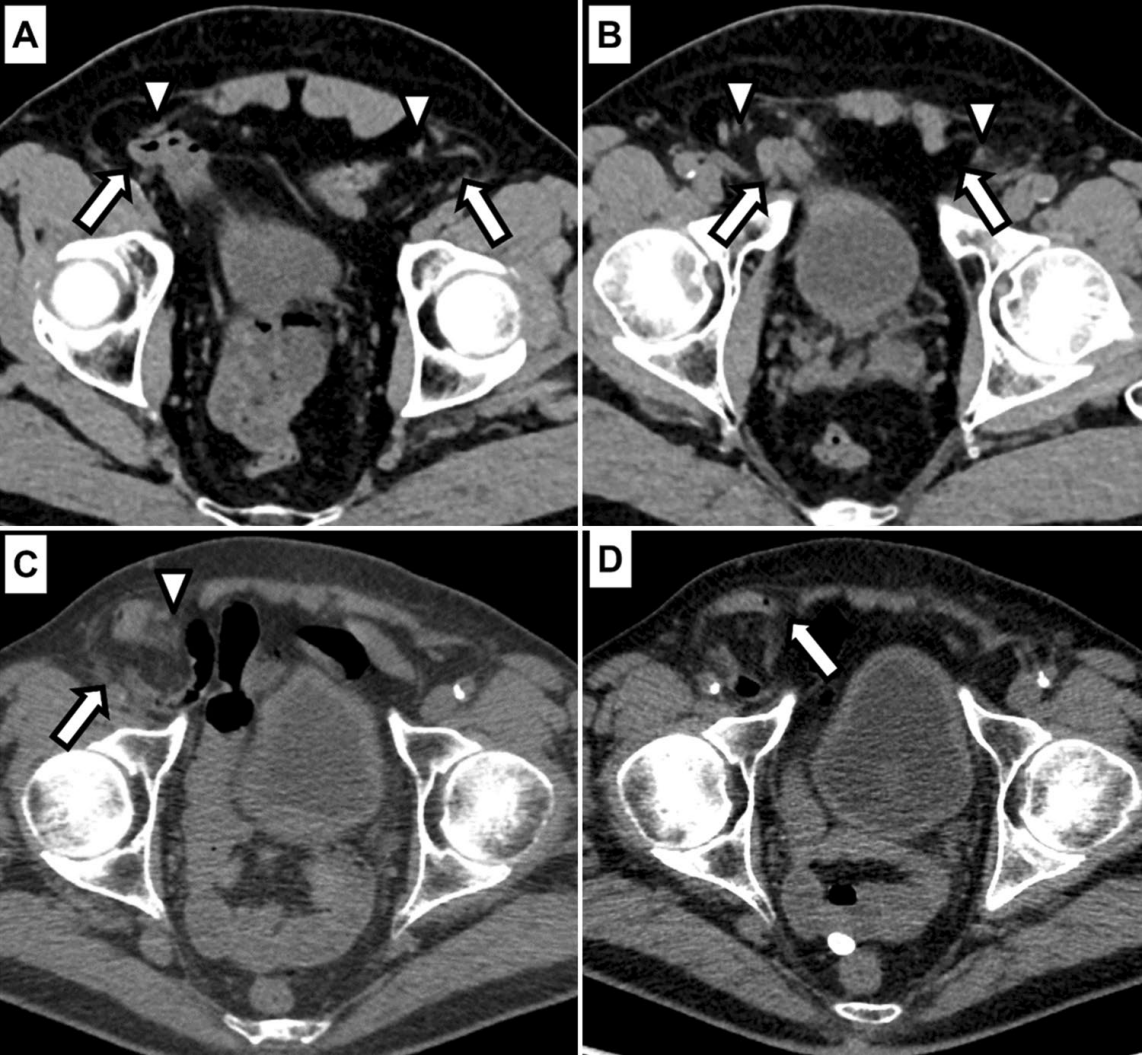
Two examples of combined inguinal hernias. Top row **A**, **B** shows axial CT images a few slices apart at the level of the groin in a patient with bilateral combined inguinal hernias. The indirect component (arrows in **A**) arises lateral to the inferior epigastric vessels (arrowhead in **A**) while the direct component (arrows in **B**) arises medial to the inferior epigastric vessels (arrowheads in **B**). This case was correctly classified by all three readers. Bottom row **C**, **D** shows axial CT images a few slices apart at the level of the groin in a patient with a right-sided combined inguinal hernia. The larger, indirect component (arrow in **C**) is seen lateral to the inferior epigastric vessels (arrowhead in **C**), but there is also a small direct component (arrow in **D**). This case was misclassified as indirect inguinal hernia by all three readers

The accuracy and balanced accuracy of Valsalva-CT for the classification of hernia type is outlined in Table 3. More details are reported in Supplemental Text 1. Figure 3 shows two examples of combined type inguinal hernias.

**Table 3:** Accuracy of Valsalva-CT for the classification of hernia type

	Reader 1	Reader 2	Reader 3
Accuracy			
All types	84.3% (80.0%, 88.0%)	77.9% (72.6%, 82.5%)	66.5% (60.5%, 72.2%)
Balanced accuracy			
Direct hernia	88.6%	82.3%	73.2%
Indirect hernia	88.6%	84.5%	74.5%
Combined hernia	74.4%	59.2%	55.0%
Correct classification*			
Direct hernia	89.5% [94/105]	88.5% [85/96]	84.9% [79/93]
Indirect hernia	89.7% [166/185]	85.2% [132/155]	69.2% [92/133]
Combined hernia	67.6% [25/37]	38.5% [10/26]	17.6% [6/34]

Numbers in parentheses represent the 95% confidence interval. Numbers in square brackets represent total numbers

^*^The denominator contains the true positive hernia cases identified by each reader. Hernia type was only classified in those cases, where readers identified a hernia

### Hernia size

The median size of the hernia neck was 19.0 mm (13.0, 25.0) for hernias that were detected by all three readers compared with 10.0 mm (8.0, 15.5) for hernias detected by only two readers. The median size of the hernia neck for cases missed by two readers was 9 mm (6.0, 10.0) compared with 7 mm (5.0, 9.0) for cases missed by all three readers. There were significant differences in neck size between cases which were correctly detected by all readers and those missed by all readers (7.0 mm [5, 9] vs. 19.0 mm [13, 24], p < 0.001) as well as for those cases detected by two readers compared with those missed by two readers [10.0 mm (8.0, 15.5) vs. 9.0 mm (6.0, 10.0), p < 0.001].

The median size of the hernia sac was 47.0 mm (35.0, 62.0 mm) for hernias that were detected by all three readers compared with 32.0 mm (20.0, 50.0 mm) for hernias detected by only two readers. The median size of the hernia sac for cases missed by two readers was 27.0 mm (17.3, 39.8) compared with 15.0 mm (12.0, 20.0) for cases missed by all three readers. There were significant differences in hernia sac size between cases which were correctly detected by all readers and those missed by all [47.0 mm (35.0, 62.0) vs. 15.0 mm (12.0, 20.0), p = 0.004], but not for those cases detected by two readers compared with those missed by two readers [32.0 mm (20.0, 50.0) vs. 27.0 mm (17.3, 39.8), p = 0.054].

The false negative rate in hernias with a neck size of < 10 mm was 33.0% (37/112), 56.3% (63/112), and 66.9% (75/112) for reader 1, reader 2, and reader 3, respectively. The false negative rate in hernias with a neck size of  ≥10 mm was 6.0% (16/269), 14.9% (40/269), and 17.1% (46/269) for reader 1, reader 2, and reader 3, respectively. Details about hernia size of combined hernias are shown in Table 1.

### Interreader agreement of Valsalva-CT for diagnosis and type of inguinal hernia

Interreader agreement for the presence of inguinal hernia between all three readers was substantial (both sides: α = 0.723, right side: α = 0.721, left side: α = 0.731) with percentage of agreement ranging from 78.8% to 85.5%. Interreader agreement was also substantial when only analyzing hernias containing more than fat (i.e., bowel, bladder) (α = 0.752) with percentage of agreement between readers ranging from 96.2%—99.0%. Interreader agreement for the type of inguinal hernia between all three readers was moderate (α = 0.522) with a percentage of agreement of 64.6%. Detailed results of interreader agreement between single readers for different subcategories are outlined in Supplemental Table 2.

## Discussion

In this study we evaluated the diagnostic performance and interreader agreement of Valsalva-CT for the detection and characterization of inguinal hernias. This study showed substantial interreader agreement (α = 0.723), high specificity (92.5–98.1%) and accuracy (81.1–91.5%) of Valsalva-CT. In contrast, sensitivity was only moderate, ranging from 68.2% to 85.8%, which was associated with missed smaller hernias.

A few studies have investigated the role of CT for the diagnosis of inguinal hernia [8, 16–19, 23–25]. However, those studies either included a small number of patients [17, 23], did not investigate the Valsalva maneuver [16, 18, 19], did not have a dedicated read-out of CT images [16], did not clearly describe their reference standard [17], or focused on differentiation between types of groin hernias [24, 25]. In a systematic review, Piga et al. [12] analyzed the existing literature on the diagnostic performance of different imaging modalities for the diagnosis of inguinal hernia. Sensitivity and specificity of CT ranged from 57 to 100% and 83 to 100%, respectively. Only one study was included where Valsalva maneuver was performed in a subset of 8 patients [26].

Jaffe et al. [17] examined CT with and without Valsalva maneuver for identifying abdominal wall hernias and found increased conspicuity of hernias with Valsalva maneuver. Notably, most cases involved abdominal wall hernias other than inguinal hernias. Furthermore, the reference standard is not clear and diagnostic performance metrics were not reported in a consistent manner. We included all surgically proven inguinal hernias, regardless of size, even those incidentally diagnosed during surgery for other hernias. Hence, our cohort also included patients with small inguinal hernias. The threshold for calling a hernia in subtle cases may vary among individual readers, which could account for the lower and more inconsistent sensitivity rates observed. This hypothesis is supported by the observation that the size of the hernia neck and sac were notably smaller in cases overlooked by all three readers, relative to those identified by all three readers.

In our study, the two radiologists who were more experienced and subspecialized in abdominal imaging had a higher accurary than the reader with experience but without subspecialization. These findings are in line with a previous study from Miller et al. [8] where a dedicated second reading of hernia scans by a radiologist led to a significant increase in accuracy compared to the baseline radiology reports (accuracy increased from 35 to 79%). Other studies have shown an increase in perceived report quality and decrease in interpretive discrepancies with subspecialized reporting [27, 28]. Subspecialized reporting can yield significant clinical advantages, particularly in instances involving small and clinically occult hernias.

Accuracy for hernia type characterization was only moderate (66.5%–84.3%). Direct and indirect inguinal hernias were classified more accurately than combined hernias. While 80% of combined hernias were recognized as a hernia, only 24% were accurately identified as a combined type. Combined hernias tend to have one component that is more pronounced than the other, potentially leading to a lower degree of accuracy in their classification [8]. Kamei et al. [18] reported correct classification in 95.8%, but with possible selection bias as only surgery was used as a reference standard and hernia size was not reported. Their data also suggests a higher misclassification rate for combined hernias. Clinically, the type of inguinal hernia may not be as important for treatment planning as the detection of any hernia or the accurate identification of hernia contents, as this may inform treatment urgency, surgical approach, and complexity [29–32]. In our study, the detection rate of inguinal hernias containing bowel or bladder was high, ranging from 94.3 to 95.2%. Only three cases (2.9%) were overlooked by all three readers.

The recurrence rates following inguinal hernia repair can be as high as 15%, and their diagnosis through clinical examination alone can prove challenging owing to scarring and fibrosis [32, 33]. Detection rates for inguinal hernias were similar between surgery-naïve patients and those with history of inguinal hernia repair in our cohort, indicating that Valsalva-CT can be used as a diagnostic tool in patients with suspected recurrent hernia.

Our study has several limitations. First, it is a single-center retrospective study with data from a tertiary referral center and hence our findings may not be representative of other clinical practices and results should be validated in a prospective multi-center setting. We tried to minimize this bias by including all patients in a consecutive manner. Nevertheless, our study includes to date the largest cohort investigating supine CT with Valsalva maneuver for the detection of inguinal hernias. Second, surgery was performed by different surgeons from our hospital and varying surgical techniques may have introduced heterogeneity. However, as opposed to reoperation rates and outcome, which are established surgeon quality metrics, intraoperative confirmation of an inguinal hernia is less likely to be affected by surgeon-level variation [34]. Furthermore, we used a composite clinical reference standard which entails the risk of over- or underestimation of the diagnostic test accuracy, can introduce verification bias, and affects the translation of our findings into a broader clinical setting. However, in the absence of a perfect reference standard we defined our reference standard based on current clinical practice guidelines for the diagnosis of inguinal hernia [5, 20]. The hernia size and European Hernia Society (EHS) groin hernia classification [5] was not consistently reported in the operative reports. Therefore, we were not able to correlate imaging-based hernia size with objective measurements from surgery in this retrospective study. Last, given the retrospective nature of the study, we were not able to compare the diagnostic accuracy of CT with other imaging modalities like US or MRI.

In conclusion, CT with Valsalva maneuver has a very high specificity and high accuracy for the diagnosis of inguinal hernia and the detection rate for inguinal hernias containing bowel or bladder is high. However, sensitivity is only moderate and affected by hernia size, as very small hernias are more frequently missed.

### Supplementary Information

Below is the link to the electronic supplementary material.

Supplementary file1 (DOCX 18 KB)

Supplementary file2 Supplemental Fig. 1: Top row shows axial (A) and sagittal (B) image of Valsalva-CT in a 61-year-old male patient. Bottom row shows axial (C) and sagittal (D) images from a CT abdomen study of the same patient without Valsalva maneuver that was done for other reasons one year prior. Note the outward bowing of the abdominal wall (arrows in A and B) due to increased abdominal pressure indicating a successful Valsalva maneuver. (TIF 4480 KB)

Supplementary file3 Supplemental Fig. 2 : A: 46-year-old male patient with previous bilateral inguinal hernia repair and surgically proven recurrent direct hernia on the right with subtle bulging of the bladder (arrow in A). Note the thin linear structure in the right groin region (arrowhead in A) representing hernia mesh from prior surgery. This subtle recurrent hernia was missed by all three readers. B: 76-year-old male patient with abdominal wall weakness and surgically proven bilateral femoral hernias and combined inguinal hernias. Note small bowel loops protruding into the wide-necked bilateral hernias. All three readers rated this case as negative for inguinal hernia and interpreted images as femoral hernias. C: 68-year-old male patient with history of radical prostatectomy presenting with a large incisional hernia in the lower midline abdominal wall and concurrent direct inguinal hernias (arrows in C) with protruding bowel loops. All three readers read this case as negative for inguinal hernia, probably due to the presence of the large incisional hernia involving the origin of the direct inguinal hernias at the Hesselbach triangle. (TIF 1322 KB)

Supplementary file4 (DOCX 14 KB)

Supplementary file5 (DOCX 14 KB)

## Data Availability

All data supporting the findings of this study are available within the paper and its supplementary material.

## Abbreviations

CI: Confidence interval
CT: Computed tomography
IQR: Interquartile range
MRI: Magnetic resonance imaging
RIS: Radiology information system
US: Ultrasound
Valsalva-CT: Computed tomography with Valsalva maneuver
