# Supplementary material for: Diagnostic performance of CT with Valsalva maneuver for the diagnosis and characterization of inguinal hernias
Source: Hernia. 2023 Jul 6;27(5):1253–61. doi: 10.1007/s10029-023-02830-y (PMC10533612; doi:10.1007/s10029-023-02830-y)
Supplement: Supplementary file 1 — Supplementary file1 (DOCX 18 KB) [file 10029_2023_2830_MOESM1_ESM.docx]

**Supplemental Text 1:**

**Diagnostic performance of Valsalva-CT for the type of inguinal hernia**

Of the 116 direct inguinal hernias, 102 (87.9%) were detected as hernias by at least two readers and 92 (79.3%) were correctly classified as the direct type by at least two readers.

Of the 215 indirect inguinal hernias, 157 (73.0%) were detected as hernias by at least two readers and 136 (63.0%) were correctly classified as the indirect type by at least two readers.

Of the 50 combined inguinal hernias, 40 (80.0%) were detected as hernias by at least two readers and 12 (24.0%) were correctly classified as the combined type by at least two readers.

**Diagnostic performance of Valsalva-CT for hernia with contents**

Of the 381 inguinal hernias, 276 (72%) contained fat only, 93 (24%) contained bowel (either small or large bowel), 11 (3%) contained parts of the bladder, and one (0.3%) contained fluid. The percentage of agreement between readers and reference standard ranged from 81.1% to 91.5% for all hernias and from 94.3 to 95.2% for hernias containing more than fat. Of those hernias containing more than fat (n=105), three cases (2.9%) were missed by all three readers (***Supplemental Figure 2***).

**Diagnostic performance of Valsalva-CT in cases with prior inguinal hernia repair**

For cases with prior inguinal hernia repair (79/351), the percentage agreement between readers and the reference standard was 91.3%, 79.1%, and 78.7% for reader 1, reader 2, and reader 3, respectively. For cases without prior inguinal hernia repair (272/351), the percentage agreement between readers and the reference standard was 91.5%, 82.3%, and 79.7% for reader 1, reader 2, and reader 3, respectively.

**Supplemental Figure 2:** A: 46-year-old male patient with previous bilateral inguinal hernia repair and surgically proven recurrent direct hernia on the right with subtle bulging of the bladder (arrow in A). Note the thin linear structure in the right groin region (arrowhead in A) representing hernia mesh from prior surgery. This subtle recurrent hernia was missed by all three readers. B: 76-year-old male patient with abdominal wall weakness and surgically proven bilateral femoral hernias and combined inguinal hernias. Note small bowel loops protruding into the wide-necked bilateral hernias. All three readers rated this case as negative for inguinal hernia and interpreted images as femoral hernias. C: 68-year-old male patient with history of radical prostatectomy presenting with a large incisional hernia in the lower midline abdominal wall and concurrent direct inguinal hernias (arrows in C) with protruding bowel loops. All three readers read this case as negative for inguinal hernia, probably due to the presence of the large incisional hernia involving the origin of the direct inguinal hernias at the Hesselbach triangle.
