# Supplementary figures and images for: Diagnostic performance of CT with Valsalva maneuver for the diagnosis and characterization of inguinal hernias
Source: Hernia. 2023 Jul 6;27(5):1253–61. doi: 10.1007/s10029-023-02830-y (PMC10533612; doi:10.1007/s10029-023-02830-y)

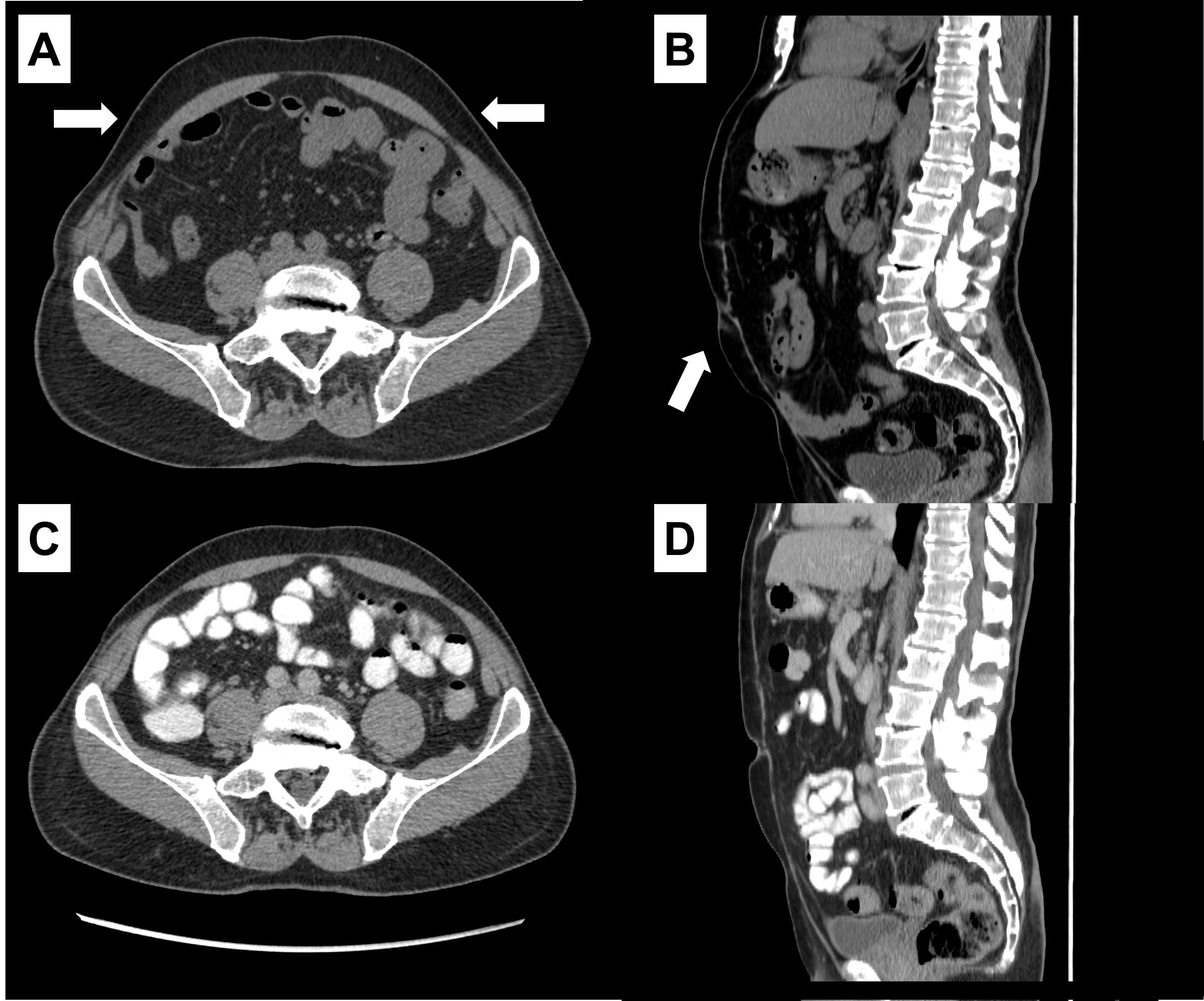

Supplement: Supplementary file 2 — Supplementary file2 Supplemental Fig. 1: Top row shows axial (A) and sagittal (B) image of Valsalva-CT in a 61-year-old male patient. Bottom row shows axial (C) and sagittal (D) images from a CT abdomen study of the same patient without Valsalva maneuver that was done for other reasons one year prior. Note the outward bowing of the abdominal wall (arrows in A and B) due to increased abdominal pressure indicating a successful Valsalva maneuver. (TIF 4480 KB) [file 10029_2023_2830_MOESM2_ESM.tif]

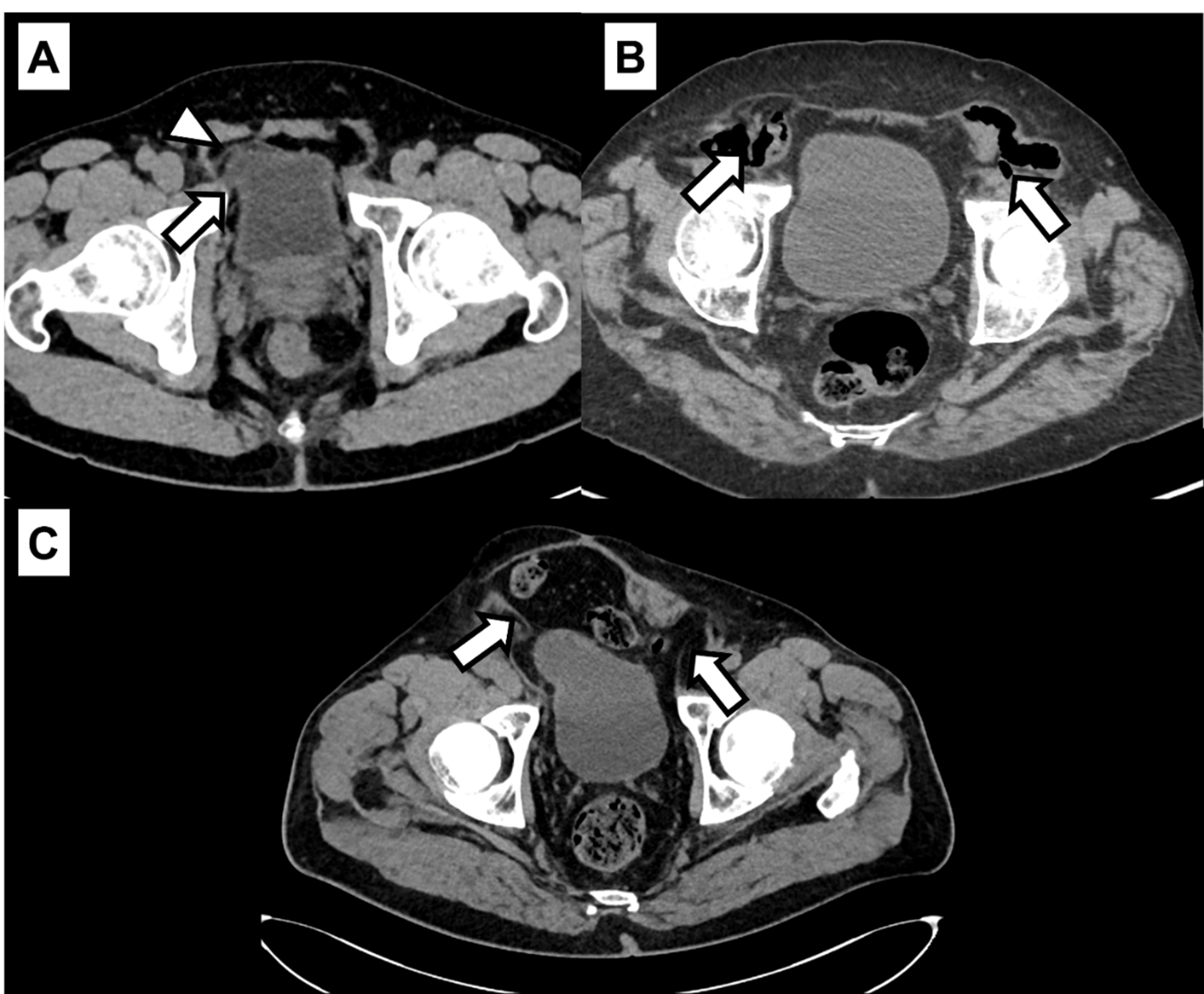

Supplement: Supplementary file 3 — Supplementary file3 Supplemental Fig. 2 : A: 46-year-old male patient with previous bilateral inguinal hernia repair and surgically proven recurrent direct hernia on the right with subtle bulging of the bladder (arrow in A). Note the thin linear structure in the right groin region (arrowhead in A) representing hernia mesh from prior surgery. This subtle recurrent hernia was missed by all three readers. B: 76-year-old male patient with abdominal wall weakness and surgically proven bilateral femoral hernias and combined inguinal hernias. Note small bowel loops protruding into the wide-necked bilateral hernias. All three readers rated this case as negative for inguinal hernia and interpreted images as femoral hernias. C: 68-year-old male patient with history of radical prostatectomy presenting with a large incisional hernia in the lower midline abdominal wall and concurrent direct inguinal hernias (arrows in C) with protruding bowel loops. All three readers read this case as negative for inguinal hernia, probably due to the presence of the large incisional hernia involving the origin of the direct inguinal hernias at the Hesselbach triangle. (TIF 1322 KB) [file 10029_2023_2830_MOESM3_ESM.tif]
