# Supplementary material for: Diagnostic performance of CT with Valsalva maneuver for the diagnosis and characterization of inguinal hernias
Source: Hernia. 2023 Jul 6;27(5):1253–61. doi: 10.1007/s10029-023-02830-y (PMC10533612; doi:10.1007/s10029-023-02830-y)
Supplement: Supplementary file 4 — Supplementary file4 (DOCX 14 KB) [file 10029_2023_2830_MOESM4_ESM.docx]

| **Scanner** | SOMATOM Force, Siemens | SOMATOM Edge Plus, Siemens |
| --- | --- | --- |
| **Tube voltage (kVp)** | 150 | 110 |
| **Reference tube current-time product (mAs)*** | 80 | 120 |
| **Slice thickness (mm)** | 2 | 2 |
| **Slice increment (mm)** | 1.5 | 1.5 |
| **Reconstruction kernel/iterative reconstruction level** | Br36d/3  (soft tissue) | Br38f/3  (soft tissue) |
| **Sections** | 2 x 192 | 128 |
| **Pitch factor** | 0.6 | 0.6 |
| **CT dose index (CTDIvol)**** | 8.3 ± 2.8 mGy | |
| **Dose-length product (DLP)**** | 407.4 ± 154.7 mGy x cm | |
| *Automated tube current modulation was used in each scan  ** Data is presented as mean ± standard deviation. | | |

**Supplemental Table 1:** Technical details of the Valsalva-CT protocols.
