# Supplementary material for: Diagnostic performance of CT with Valsalva maneuver for the diagnosis and characterization of inguinal hernias
Source: Hernia. 2023 Jul 6;27(5):1253–61. doi: 10.1007/s10029-023-02830-y (PMC10533612; doi:10.1007/s10029-023-02830-y)
Supplement: Supplementary file 5 — Supplementary file5 (DOCX 14 KB) [file 10029_2023_2830_MOESM5_ESM.docx]

**Supplemental Table 2:** Interreader agreement (Krippendorff’s alpha) and percentage of agreement between the three readers.

|  | **All inguinal hernias** | **Inguinal hernias**  **with contents*** | **Hernia type** | **Surgery-naïve**** | **History of inguinal hernia repair***** |
| --- | --- | --- | --- | --- | --- |
| **Krippendorff’s α coefficient** |  |  |  |  |  |
| **Reader 1 vs. Reader 2** | 0.707 | 0.648 | 0.663 | 0.707 | 0.706 |
| **Reader 1 vs. Reader 3** | 0.718 | 0.905 | 0.421 | 0.743 | 0.713 |
| **Reader 2 vs. Reader 3** | 0.744 | 0.714 | 0.479 | 0.778 | 0.737 |
| **Percentage agreement** |  |  |  |  |  |
| **Reader 1 vs. Reader 2** | 85.5% | 96.2% | 84.0% | 85.3% | 86.1% |
| **Reader 1 vs. Reader 3** | 86.2% | 99.0% | 70.5% | 85.9% | 87.8% |
| **Reader 2 vs. Reader 3** | 87.6% | 97.1% | 72.7% | 87.2% | 89.6% |
| * Only hernias containing more than fat only (e.g., small bowel, large bowel, bladder, etc.)  ** Only cases without prior inguinal hernia repair  *** Only cases with prior inguinal hernia repair | | | | | |
